# Influence of age, gender, and willingness to adopt former foodstuffs on the perception of Italian farm animal veterinarians

**DOI:** 10.3389/fvets.2024.1396807

**Published:** 2024-06-06

**Authors:** Elena Diaz Vicuna, Karthika Srikanthithasan, Rosangela Odore, Stefano Massaglia, Valentina Maria Merlino, Andrea Giorgino, Laura Ozella, Achille Schiavone, Francesca Romana Massacci, Jatziri Mota-Gutierrez, Claudio Forte

**Affiliations:** ^1^Dipartimento di Scienze Veterinarie, Università di Torino, Turin, Italy; ^2^Dipartimento di Scienze Agrarie, Forestali e Alimentari, Università di Torino, Turin, Italy; ^3^Istituto Zooprofilattico Sperimentale dell’Umbria e delle Marche “Togo Rosati”, Perugia, Italy

**Keywords:** safety perceptions, feedstuffs, alternative feed, circular economy, sustainability

## Abstract

**Background:**

Veterinarians play an essential role in improving animal care, as they are often viewed as trusted advisors, particularly in relation to disease control and management; however, little is known about veterinarians’ perceptions and attitudes toward alternative feeds. The aim of this study was to investigate the influence of age, gender, and willingness to adopt on the attitudes of livestock veterinarians toward the use of alternative feeds in farm animals.

**Methods:**

A total of 136 active veterinarians completed the online survey, distributed through the main veterinary associations in Italy. The questionnaire contained items on dietary recommendation, awareness, benefit and safety perceptions, and a willingness to adopt former foodstuffs (FFs), complemented with socio-demographic questions.

**Results:**

Almost 90% of the population reported a willingness to adopt FFs as feed. Men and women did not share the same perceptions of the nutritional composition of FFs, while the importance of product availability was found to be a key factor driving the age difference. Participants willing to adopt FFs as feed linked positive attitudes to attributes such as digestibility, energy intake, and positive social implications.

**Conclusion:**

Our findings provide a basic background on the current use of the FFs in Italy and suggest the need for the development of educational programs and marketing strategies to enhance the acceptability of FFs in farm animals to ultimately promote the transition toward more sustainable animal production. This study has limitations, including the number of recorded responses and reliance on national estimates. Future research is needed to investigate the perceptions of farmers and animal nutritionist from different countries. This could provide a more detailed picture of the current situation in Europe about the potential of using FFs in farm animals’ feed, thus further contributing toward a greener and safer livestock production sector.

## 1 Introduction

Animal feed plays a crucial role in determining the sustainability performance of animal production systems. The choice of diet affects the animal production chain downstream on, for example, greenhouse gas (GHG) emissions, animal productivity, animal health, and product safety and quality (1). In this regard, the Food and Agriculture Organization seeks to assess and minimize the negative effects of animal diets on sustainability by informing changes in practices among farmers, farmer organizations, and the feed industry (2). The FAO (2) report showed that sustainability indicators influenced actors in the animal production chain on dimensions like the planet (water pollution, deforestation), people (affordability, competition with human food), and profit (socio-environmental costs, benefit–cost ratio).

Incorporating alternative ingredients into animal diets represents an effective strategy benefitting both the environment and the animal sector in shaping sustainable feed solutions (3). Currently, innovative raw materials for animal feed are being explored; among the most promising findings, agro-industrial co-products and by-products, food leftovers, and former foodstuffs (FFs) are gaining more and more attention (4–9). FFs are defined by the Commission Regulation (EU) 1104/2022 (10) as *“foodstuffs, […] manufactured for human consumption in full compliance with the EU food law, but which are no longer intended for human consumption […] and which do not present any health risks when used as feed.”* The integration of FFs into farm animal rations can reduce the farmers’ reliance on cereal grains, oils, and sugars while improving nutritional efficiency, thus contributing to a more sustainable food chain by minimizing waste and promoting the use of circular ingredients in feed (11).

From the nutritional point of view, existing literature indicates that FFs are rich in carbohydrates and fats, with varying levels based on their origin. They are therefore characterized by a highly energetic content, valuable for animal feed (7). According to Giromini et al. (5), FFs share a nutritional composition similar to wheat grain but with a higher energy content, primarily contributed by fats and starch, and also exhibit high digestibility. Raising FFs’ awareness in animal feed can bring numerous benefits such as economic, ecological, and ethical considerations (11). From the economic point of view, this alternative feed material can lead to a cost-effective replacement to traditional ingredients while diversifying the farmers’ feed sources, thus reducing their reliance on ingredients often subjected to price fluctuations due to the current market’s high volatility (12, 13). FFs also provide significant ecological advantages that play a crucial role in fostering a sustainable and ethical food system. These benefits encompass the reduction of food waste, enhanced resource efficiency and lowered GHG emissions (4).

From a legislative perspective, feed ingredients approved for use in food-producing livestock are regularly updated by the European Commission in the Catalogue of feed materials (14). FFs were introduced to this list with Reg. UE 68/2013 (15) after critical safety evaluations to minimize main hazards related to their employment in animal nutrition (7, 16). However, to foster the acceptance and integration of FFs in animal feed, it is imperative to actively challenge the prevailing perception that views FFs as mere garbage (9).

Introducing sustainable alternative materials into feed requires the possession of knowledge about these practices by all the main figures working in the zootechnical field, such as farmers, veterinarians, technicians, the feed manufacturing industry, and feed legislation. The adoption of new agricultural technologies by farmers depends on various factors, with their perceptions playing a crucial role (17). Moreover, the role of vets and veterinary technicians is crucial to the progression toward more sustainable livestock farming, although their significance is not widely acknowledged in public perception (18). To maintain their relevance in society, veterinarians must prioritize addressing climate change (19), especially in response to the increasing environmental concerns. Currently, veterinarians are actively promoting sustainability in their communities by tackling waste reduction and exploring sustainable practices in the livestock sector (20). Both veterinary students and professionals are keen to adopt environmentally sustainable practices in their field. To achieve this, accessible and evidence-based sustainability strategies relevant to the veterinary field are essential, with consideration given to staff attitudes and organizational behaviors. Training, education, and personalized environmental goals at individual level prove effective, while group problem-solving is encouraged through collective incentives. By aligning with personal values and emphasizing long-term benefits, veterinary practices can successfully implement sustainable changes, benefiting both the environment and the economy while supporting the wellbeing of veterinarians and clients (19, 21, 22).

Understanding the impact of environmental aspects and demographic factors on perspectives regarding the livestock industry is a significant concern. Recognizing the interactions of these socio-demographic traits with individual convictions and professional backgrounds offers valuable insights into the varied stances of veterinarians on sustainable livestock production (23, 24). Addressing this diversity of viewpoints could enhance sustainability initiatives within the veterinary field. However, it’s worth noting that the potential contribution of veterinarians as stakeholders in various environmentally friendly farming practices, particularly alternative feeding methods like FFs, has not yet been fully recognized.

Therefore, understanding vets’ knowledge and perceptions is essential for excellence and competitive advantage, especially in innovative products like feed alternatives. Recent studies have shown the importance to address the perceptions and attitudes toward feed materials from the main figures involved in livestock farming, such as animal breeders, food and feed processors, and veterinarians, to understand their knowledge and needs (20, 25, 26). Education campaigns focusing in raising awareness about the properties of FFs and their positive environmental impact might help animal professionals to make informed decisions about which industrial feed to use (9). To bridge this gap, the present study aims to investigate the influence of demographic information and willingness to adopt on the perceptions of Italian farm animal veterinarians’ perceptions toward the use of innovative feed raw materials like FFs as promoters of environmentally friendly practices.

## 2 Materials and methods

### 2.1 Participants and data collection

Farm animal veterinarians were selected to be the main targets of this survey, as they play a crucial role in the farm animal sector. Recruitment was performed by requesting the main veterinary associations in Italy to distribute the web-based questionnaire with request for disclosure to the members. A total of 157 participants completed the survey. The inclusion criteria were: active professional practice in Italy for at least 2 years, direct participation to the diets’ planning (feed composition, origin, and quality of raw materials), and/or involvement in the feed production process (evaluation of the raw materials’ quality, monitoring of the main production phases and check of the final product’s quality) of farm animals. Professionals exclusively working as small animal veterinarians, food safety and inspection veterinarians, or research veterinarians were excluded (*n* = 21). Responses from veterinarians specialized in small animal, working in public health, or in the research field were removed as the responses were not representative to the larger population. In addition to the best of the author’s knowledge, there is scarce literature studying farm animal vets’ perceptions, contrary to the other types of specialization, small animal veterinarians in particular (27, 28). Only people who agreed to participate by giving their consent for data usage were included.

A survey of active veterinarians was conducted between March and September 2022. Participants filled in the survey anonymously and voluntary and did not receive monetary compensation for their participation. This study follows the ethical standard defined by the Declaration of Helsinki and approved by the Ethical Committee of the Department of Veterinary Sciences from the University of Turin, approval n.01698737.

### 2.2 Questionnaire

The questionnaire was structured into 21 compulsory, close-ended questions, divided into four main sections: (1) socio-demographics, (2) FFs dietary recommendation and awareness, (3) perceptions, and (4) willingness to adopt (WTA). The initial section started with an introductive paragraph structured to allow respondents to contextualize the survey while ensuring not to influence their responses. The text provided the participants with a brief overview of FFs and related legislative framework, the list of inclusion/exclusion criteria for participation, and the aim of the survey (“our intention is to verify on multiple levels the perception and knowledge of a product from the feed industry which is gaining ever greater interest”). Next, participants were asked six questions concerning their demographic profile, including gender, age, geographical location, and number of years of experience in animal management, type of animal species handled (see Supplementary Appendix).

In the second section, participants were asked whether they recommend the use of FF as feed or not through the employment of a multiple-choice question (“Yes/No/Not Sure”). Based on the answers provided, respondents were categorized as pro-FFs, con-FFs, and uncertain, respectively. Next, participants were asked to select what are FFs products among the following options: (a) “Co-product of the agri-food supply chain whose production is impossible to avoid, but which has gained greater economic value (e.g., wheat and bran)” (29); (b) “Food product no longer intended for human consumption due to non-compliance of an aesthetic-commercial nature” (7); (c) “Waste generated during the production process” (7); (d) “By-product unintentionally generated during the production process, characterized by commercial value (bran and distiller)” (30). When participants correctly answered the FFs’ definition, they were grouped as “high awareness,” and when they answered incorrectly, they were grouped as “low awareness.”

In the third section, participants were then asked to express their importance of nine attributes for FFs, namely: (1) economic advantage; (2) feed consistency; (3) environmental sustainability; (4) positive social implications; (5) product availability; (6) antioxidant properties; (7) vitamin content; (8) supply of by-pass protein; and (9) digestibility and energy intake of FFs using a 5-point Likert scale (1 = not at all important to 5 = very important) (31, 32). In addition, participants were asked about their perception of FFs’ safety, considering the three main hazards most typically associated with FFs in literature namely, microbiological risk, toxicological risk, and inaccuracy between actual and declared values reported on the label employing a single, close-ended question (15, 33, 34). Finally, in the last section, participants were asked about their WTA feed products obtained from FFs through a single, close-ended question.

### 2.3 Statistical analysis

A comparison of mean scores between the level of importance of FFs perceptions (measured on a 5-point scale as interval variables), and level of knowledge of FFs safety (dummy variable: yes or no) as feed according to age, gender, FFs awareness, dietary recommendation, and willingness to adopt was assessed using analysis of variance (ANOVA). Statistical analyses were carried out using generalized linear mixed-effect models (GLMMs). Generalized linear mixed models (GLMMs) have been formulated to correct the assumptions made in linear mixed models, such as the straight relationship between some known function of the mean of *y* and the predictors *x* and random effects *z* (assumption check: plotting residual plots); constant variance (Levene’s test: *p*-value less than 0.05) and that random effects follow a normal distribution (Shapiro–Wilk test: *p*-value greater than 0.05). The assumptions that were met were (1) the observed *y* are independent, conditional on some predictors *x* (random sampling); (2) the response *y* comes from a known distribution from the exponential family, with a known mean variance relationship (residual plots); (3) random effects *z* are independent of *y* (random sampling). Mixed models were chosen because of their ability to capture both fixed (Gender: women and men; Age: young adults, middle-aged adults, and older adults; Awareness: high and low; Dietary recommendation: pro-FFs, uncertain, and con-FFs; and WTA: willing and unwilling, Type of species management: ruminants, poultry, swine, and other, were added to the GLMM as covariates) and random effects (number of subjects, *n* = 136). Power calculations for the sample size was used to ensure a significance level = 0.05 and *f* values = 0.4, using the “*pwr”* function (power = 0.98). The *p*-values were adjusted using Bonferroni’s method, and when the mixed model revealed significant differences (*p* < 0.05), the least significant difference test was applied. Mixed models were built and evaluated according to Crawley (35) using R version 3.3.2. Potential confounding variables that could influence the results of the present study include the fact that veterinarians willing to adopt former foodstuff may have clients who inquire more about natural foods and products compared with vets unwilling to adopt. Additionally, factors such as educational level, rural or urban upbringing, and country of origin of respondents may also play a role. On the other hand, correlation analysis (Spearman correlation) between the perceptions of the use of FFs in farm animals and willingness to adopt FFs as feed was conducted. Spearman’s rank correlation coefficient was obtained as a measure of the association between the perceptions toward FFs and willingness to adopt using the “*psych*” function and plotted through the “*corrplot*” package of R.

## 3 Results

The socio-demographic characteristics of the sample are described in Table 1. Briefly, the present survey was completed by 43 women and 93 men aged between 18 and 62 years from different regions of Italy (Table 1). More than 50% of the women population is aged between 31 and 50 years old, while more than 50% of the men population is over 51 years old. The average years of experience in working with animals is 15 years for women and 25 years for men. Almost more than 50% of respondents are aware of the definition of FFs but do not currently recommend it. More than 90% of respondents are willing to adopt FFs as feed as shown in Table 1.

**Table 1:** Socio-demographic characteristics of the surveyed Italian veterinarians.

Socio-demographics		
Gender	Women	Men
	*n* = 43	*n* = 93
Socio-economics		
*Age*		
∙ Young adults (18–31 years)	11.63%	9.68%
∙ Middle-aged adults (31–50 years)	65.12%	30.11%
∙ Older adults (51 and more year)	23.26%	60.22%
*Region*		
∙ North	46.51%	54.84%
∙ Centre	41.86%	29.03%
∙ South and Islands	11.63%	15.05%
*Average years of animal experience*	15	25
Former foodstuffs awareness		
∙ High awareness	65.12%	48.39%
∙ Low awareness	34.88%	51.61%
Willingness to adopt former foodstuffs		
∙ Willing	93.02%	91.40%
∙ Unwilling	6.98%	8.60%
Dietary recommendations		
∙ Con-FFs	60.47%	53.76%
∙ Uncertain	27.91%	18.28%
∙ Pro-FFs	11.63%	27.96%
Species		
∙ Poultry	13.95%	15.05%
∙ Ruminants	58.14%	50.54%
∙ Swine	9.30%	10.75%
∙ Other	18.60%	23.66%

### 3.1 Italian farm animal vets’ perceptions of the use of FFs in farm animals according to age, willingness to adopt and dietary recommendation

Overall, all veterinarians did not consider the antioxidant properties, vitamin content, and by-pass protein supply of FFs to be important (perception scores below 3.4, Table 2). In contrast, the general product characteristics, such as economic advantage, feed consistency, environmental sustainability, and product availability were indicated as important aspects of FFs (perception scores higher than 3.4, Table 2).

**Table 2:** Effect of gender in the perception of the use of former foodstuffs in farm animals among surveyed Italian veterinarians (mean and standard error scores, *n* = 136).

	Women		Men		*P*-value	*F*-value
	Mean	SE	Mean	SE		
Vitamin content	2.79	0.27a	2.26	0.22b	**0.003**	**9.0609**
Supply of by-pass protein	2.90	0.26	2.58	0.22	0.110	2.5948
Antioxidant properties	3.08	0.28a	2.36	0.24b	**0.001**	**11.7226**
Positive social implication	3.13	0.34	3.23	0.28	0.763	0.0912
Feed consistency	3.15	0.34	3.69	0.28	0.100	2.7524
Economic advantage	3.45	0.30	3.57	0.25	0.087	0.2137
Digestibility and energy intake	3.47	0.23	3.39	0.19	0.671	2.5948
Product availability	3.58	0.30	3.88	0.25	0.441	0.5972
Environmental sustainability	3.77	0.34	3.68	0.29	0.964	0.0021

Scales: 5 = Very important; 4 = Slightly important, 3 = Neutral, 2 = Slightly unimportant; 1 = Not important at all. Abbreviations. SE = Standard error. Different letters indicate statistical difference related to perceptions ranking using least significant difference test (*p* < 0.05). *p*-values were adjusted using Bonferroni’s method. Bold values indicate statistical differences related to perceptions ranking using least significant differences test (*p* < 0 .05).

The analysis of the relationship between the perceptions of using FFs in farm animals and gender, age, willingness to adopt, and dietary recommendation category significantly differentiated the perception of farm animals’ veterinarians. In detail, women considered more important the vitamin content (*p* = 0.001) and antioxidant properties (*p* = 0.003) of FFs than men (Table 2). The importance of digestibility and energy intake (*p* = 0.022) and positive social implications (*p* = 0.037) was more positive in participants willing to adopt than the unwilling ones (Table 3). Additionally, participants labeled as “uncertain” reported more important the economic advantage (*p* < 0.0001), environmental sustainability (*p* = 0.832), positive social implications (*p* = 0.003), and supply of by-pass protein of FFs (*p* = 0.043) compared with both pro-FFs and con-FFs (Table 4). Regarding the age effect, young adults reported more important (*p* = 0.043) the product availability than older adults (Table 5). In contrast, the type of species managed and FFs awareness did not significantly differ in any item.

**Table 3:** Effect of willingness to adopt former foodstuffs and the perceived effects of their use in fam animals’ nutrition among surveyed Italian veterinarians (mean and standard error scores, *n* = 136).

	Willing to adopt		Unwilling to adopt		*P*-value	*F*-value
	Mean	SE	Mean	SE		
Antioxidant properties	2.70	0.16	2.75	0.39	0.900	0.0158
Vitamin content	2.76	0.15	2.29	0.37	0.181	1.8066
Supply of by-pass protein	3.04	0.15	2.43	0.36	0.072	3.2933
Feed consistency	3.38	0.19	3.47	0.46	0.833	0.1846
Positive social implications	3.64	0.19a	2.72	0.46b	**0.037**	**4.4270**
Digestibility and energy intake	3.77	0.13a	3.09	0.32b	**0.022**	**3.2933**
Economic advantage	3.84	0.17	3.18	0.41	0.645	2.9842
Environmental sustainability	3.90	0.19	3.55	0.47	0.436	0.6109
Product availability	3.96	0.17	3.50	0.41	0.240	1.3926

Scales: 5 = Very important; 4 = Slightly important, 3 = Neutral, 2 = Slightly unimportant; 1 = Not important at all. SE = Standard error. Different letters indicate statistical difference related to perceptions ranking using least significant difference test (*P* < 0.05). *P*-values were adjusted using Bonferroni’s method. Bold values indicate statistical differences related to perceptions ranking using least significant differences test (*P* < 0 .05).

**Table 4:** Dietary recommendation difference in the level of importance of the perceptions of the use of former foodstuffs in farm animals among surveyed Italian veterinarians (mean and standard error scores, *n* = 136).

	Pro-FFs		Uncertain		Con-FFs		*P*-value	*F*-value
	Mean	SE	Mean	SE	Mean	SE		
Supply of by-pass protein	2.40	0.29b	2.98	0.26a	2.84	0.21a	**0.043**	**3.2259**
Vitamin content	2.45	0.30	2.44	0.27	2.68	0.22	0.362	1.0257
Antioxidant properties	2.85	0.29	2.54	0.31	2.79	0.23	0.104	2.3061
Positive social implications	3.30	0.38ab	3.53	0.34a	2.70	0.27b	**0.003**	**6.0798**
Feed consistency	3.45	0.38	3.43	0.34	3.39	0.27	0.832	0.0446
Digestibility and energy intake	3.64	0.26	3.45	0.23	3.21	0.19	0.077	3.2259
Environmental sustainability	3.77	0.38ab	4.31	0.35a	3.10	0.28b	**0.000**	**9.4239**
Economic advantage	3.80	0.33a	3.87	0.30a	2.86	0.24b	**<0.0001**	**12.8155**
Product availability	3.82	0.33	3.88	0.30	3.49	0.24	0.185	1.7134

Scales: 5 = Very important; 4 = Slightly important, 3 = Neutral, 2 = Slightly unimportant; 1 = Not important at all. SE = Standard error. Different letters indicate statistical difference related to perceptions ranking using least significant difference test (*P* < 0.05). *P*-values were adjusted using Bonferroni’s method. Bold values indicate statistical differences related to perceptions ranking using least significant differences test (*P* < 0 .05).

**Table 5:** Age difference in the level of importance of the perceptions of the use of former foodstuffs in farm animals among surveyed Italian veterinarians (mean and standard error scores, *n* = 136).

	Young		Middle-aged		Old adults		*P*-value	*F*-value
	Mean	SE	Mean	SE	Mean	SE		
Vitamin content	2.66	0.37	2.53	0.22	2.38	0.22	0.524	0.6497
Positive social implications	2.93	0.46	3.32	0.27	3.29	0.28	0.784	0.2443
Supply of by-pass protein	2.95	0.36	2.63	0.21	2.63	0.22	0.409	0.9009
Antioxidant properties	3.13	0.39	2.50	0.23	2.54	0.24	0.169	1.8038
Feed consistency	3.38	0.46	3.58	0.27	3.31	0.28	0.586	0.5374
Economic advantage	3.46	0.40	3.57	0.24	3.50	0.25	0.947	0.0545
Environmental sustainability	3.52	0.47	3.75	0.28	3.91	0.29	0.654	0.4263
Digestibility and energy intake	3.63	0.32	3.24	0.19	3.41	0.19	0.212	0.9009
Product availability	4.19	0.41a	3.64	0.24ab	3.36	0.25b	**0.043**	**3.2298**

Scales: 5 = Very important; 4 = Slightly important. 3 = Neutral. 2 = Slightly unimportant; 1 = Not important at all. SE = Standard error. Different letters indicate statistical difference related to risk perception ranking using least significant difference test (*P* < 0.05). *P*-values were adjusted using Bonferroni’s method. Bold values indicate statistical differences related to perceptions ranking using least significant differences test (*P* < 0 .05).

No significant differences were found between the knowledge level of the main risks associated with FFs and the various demographic factors (age, gender, willingness to adopt, type of species managed, level awareness or dietary recommendation; data not shown).

### 3.2 The influence of Italian farm animal vets’ benefit perceptions and willingness to adopt former foodstuffs in farm animals

**Figure 1:**
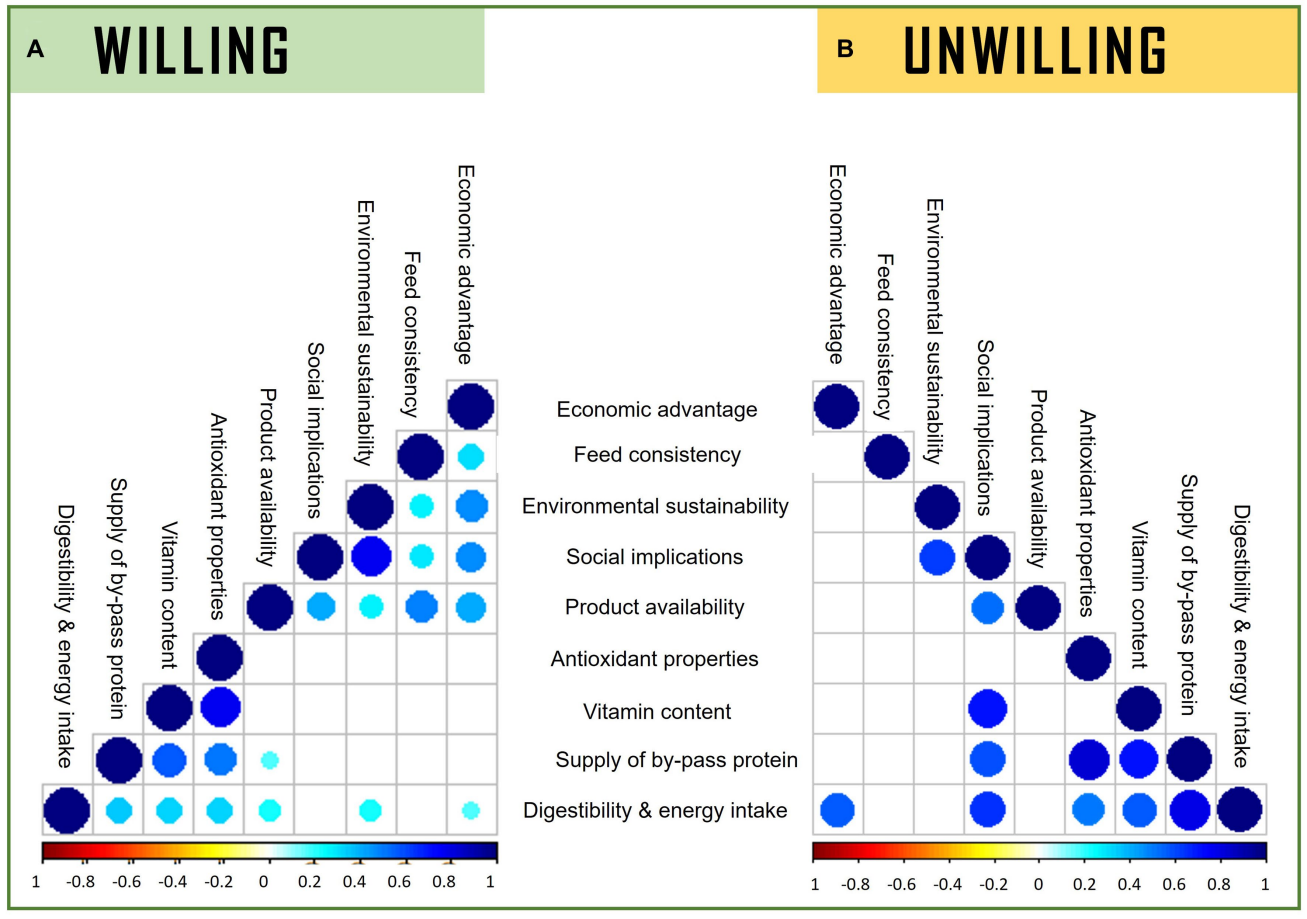
Correlation plot illustrates Spearman’s correlation between the level of importance of the perceptions of the use of former foodstuffs in farm animals and willingness to adopt among surveyed Italian veterinarians. Figures are labeled according to willingness of participants to adopt FFs as feed **(A)** Willing to adopt and **(B)** Unwilling to adopt. Only significant associations between perceptions are only shown (*p* < 0.05). The intensity of the colors represents the degree of correlation between the perceptions, as measured by the Spearman’s correlation, where the blue color represents a positive degree of correlation and the red one a negative correlation.

A Spearman correlation test was conducted to examine the relationship between the intention to adopt FFs as feed in the future and farm animal veterinarians’ perceptions of the benefits of using FFs in farm animals. The results revealed a positive correlation among feed consistency, environmental sustainability, positive social implications, economic advantage, product availability, and various nutritional aspects (vitamin content, antioxidant properties, supply of by-pass protein, and digestibility and energy intake). These correlations are illustrated in Figure 1, with corresponding rho and *p*-values reported in Supplementary Table S1. Fewer different correlations were observed in vets unwilling to adopt FFs as feed (Figure 1).

Moreover, the importance of social implications was found to be positively correlated with the importance of vitamin content, supply of by-pass protein, digestibility and energy intake, product availability, and environmental sustainability of using FFs as feed (rho = 0.31, 0.60, 0.65, 0.53, 0.62, respectively), as shown in Figure 1. The corresponding rho and *p*-values are reported in Supplementary Table S1.

## 4 Discussion

The level of knowledge and perceptions regarding the existing feed ingredients, strategies, and systems of different stakeholders are crucial for enhancing feed sustainability (36). Despite the significant role of veterinarians in ensuring balanced, safe, and nutritive diets, to the best of authors’ knowledge, existing literature has not investigated the vets’ perceptions toward the use of FFs in farm animals. Therefore, the objective of this study was to explore how socio-demographic factors, WTA, and current dietary recommendations affect the perceptions of Italian farm animal veterinarians regarding the utilization of FFs in farm animals.

The findings of this study suggest that approximately 50% of vets are aware of FFs’ definition but are not currently recommending them. This outcome aligns with Luciano et al. (37), who reported that the utilization of alternative feeds for farm animals in Europe is still in its early stages. However, more than 90% of respondents answered to be willing to adopt FFs, which is in accordance with future expectations predicting an increase in FFs’ use as feed due to their higher economic advantage, environmental sustainability, and ethical benefits compared with traditional feeds (38). The trend toward the utilization of FFs, particularly driven by pro-FFs veterinarians as observed in the present study, supports the assumption that the use of FFs in Europe may increase in the future. However, further research is necessary to identify the technological, economic, institutional, and human-specific factors that specifically contribute to the adoption of alternative feeds.

In relation to the gender effect on the perceptions of FFs’ characteristics, women considered the vitamin content and antioxidant properties of FFs to be more important than men. Studies evaluating the consumers’ perspective when purchasing different types of food products report that this gender-gap can be attributed to the tendency of women to prefer “healthier foods” (39, 40). Regarding the age effect, the present study suggested that product availability was more relevant to “younger” participants than to “older” ones. An explanation to this could be due to the growing attention toward animal nutrition only in recent years, thus leading to a more pronounced interest in younger professionals than their older counterparts (41). However, while many studies evaluating the acceptance of alternative feeds tend to focus on assessing consumers’ opinion on the final product, there is a notable gap in analyzing the perceptions of the stakeholders (42). Therefore, efforts aimed at identifying the key factors driving preferences for alternative feedstuffs among these professionals are crucial for developing appropriate marketing strategies and coherent educational programs.

Farm animal vets unwilling to adopt FFs were characterized by a general lack of knowledge and disinterest toward the economic advantage, environmental sustainability, positive social implications, and supply of by-pass protein characteristics of FFs. Existing literature indicates an overall uninterest in products “uncommon” in agricultural and animal practices (43), variable levels of trust in the reliability of the values reported on the nutritional label (44), and high level of sensitivity toward the composition of alternative feeds (insect meal in aquaculture feeding) (45). The findings of the present study align with existing literature which identifies market availability (42) and economic impact as the limiting factors for the use of alternative feeds (46). In relation to the environmental sustainability, there is limited research on alternative and sustainable feed options in the veterinarian sector (47). The limited existing literature on the matter indicates a high level of interest in environmental sustainability from different stakeholders and highlights the lack of educational programs on the subject at both undergraduate and post-graduate level (22, 48–50).

Although the significance of the “social factor” in the food and feed supply chain is rarely investigated (51, 52), the present study suggests that respondents’ considered important to adopt FFs in farm animals by linking it to an improved social implication. An unveiled ambivalence regarding sustainable management for veterinarians was associated with an economic aspect (53). Research suggests that greater profitability for veterinarians’ clients would have led to the creation of higher income for their practice (53). These findings suggest that the interest showed by the respondents of this study was also driven by the interest to provide their clients with a more economically advantageous product. Veterinarians’ interest in the sustainability factor might be correlated with the economic aspect, as suggested by Kramer et al. (54), who reported the veterinarians’ concerns about climate change, especially in terms of its the economic impacts in relation to the animals. Considering that feed typically represents one of the main costs for the animals’ management, this finding appears applicable for the results of the present study (55). However, it is important to highlight that veterinarians need to engage more in ethical discussions to ensure animal welfare and animal suffering (56). Further investigation into the moral footprint of animal products in the eyes of consumers and stakeholders is imperative due to the significant, yet often undervalued, impact of this element (57).

## 5 Limitations and implications

This study has strengths and weakness. Motives for and against participation could be possible starting points for approaches to overcome recruitment difficulties. Inquiring about the willing to adopt FFs might have caused participants to report higher willingness levels than the effective ones. Additionally, using a “Yes/No/Unsure” answers rather than a Likert scale to investigate on the willingness to adopt FFs as feed could have led to responses bias. However, this format was chosen to enhance clarity (binary responses reduce the risk of confusion or misunderstanding), lower tendency for neutrality (participants have to choose between “yes” and “no,” which could lead to more thoughtful responses and greater clarity about opinions), and lower likelihood of measurement error.

The response of a total of 136 farm animal veterinarians from one country is relatively low and provides only a small representation of the European context. However, our findings represent the voice of key stakeholders influencing sustainable feed ingredients and describe the reality of one of the leading members of the European Union. In addition to the small sample size, the methodology used of this study has potential limitations, such as no exclusion criteria concerning the quality of answers. They are therefore subject to bias. To address this limitation, the use of methodological strategies such as randomized controlled trials, or mixed methods, as well as the use of online survey companies is highly advised to prompt access to a broad audience, wider geographic reach, high response rates, low straight lining, and strengthen the validity of the results.

On the other hand, our inclusion criteria can be considered a strength. The survey was conducted online, allowing participants to respond at their own pace and in a private setting to encourage honest responses. Importantly, there are no similar studies conducted in other European countries targeting professionals working within the animal farming system. In fact, most research evaluates the perception of final consumers of the food production animals fed with alternative feeds, such as microalgae, insects, and biofuel co-products (58–60).

The lack of interest on the use of FFs in farm animals observed in the present study could represent a barrier toward a greener, safer alternative to traditional feeds. Future research is needed to better understand the overall situation of the current situation in Europe about the possibility of using FFs in farm animals’ feeds. In particular, differences based on the type of animal species managed and the perception of other stakeholders involved in the zootechnical field, such as farmers, animal nutritionist, and operators of the feed manufacturing industry, should be investigated. This is crucial due to the highly varied landscape characterizing the sector in Europe (61, 62). Comparing the perceptions of different animal nutrition stakeholders and examining their effects on each other, along with evaluating the perceptions of final consumers, could provide a solid foundation for developing further marketing strategies for FFs. Additionally, raising awareness about the use of alternative feeds and other sustainable solutions in veterinary practice, through both undergraduate and postgraduate programs, could help shift attitudes away from hostility toward new technologies and solutions, toward a more sustainable livestock sector (22, 54, 63). Further research is necessary to assess the effectiveness of food policies and interventions in promoting behavioral change and sustainability, ensuring that policies, programs, projects, and initiatives achieve their intended purpose.

## 6 Conclusion

Understanding which factors could influence farm animal veterinarians to adopt FFs plays a key role in the prospect of sustainable feed. While the findings of the present study are limited by a reduced participation of veterinarians, it appears that FFs are not widely recommended as feed by the majority of the population studied. However, our results demonstrate that the use of FFs in animal feed in Italy is of interest to the involved veterinarians and could therefore assume a primary role in the sector in future years. The implications of these findings include the need to establish dietary guidelines for the use of FFs as feed ingredients and the development of an official quality certification. This would enable FFs’ producers to offer a more reliable product to stakeholders, who in turn would be more willing to try this product. Additionally, it is imperative to provide theoretical courses related to the sustainability of the livestock sector for all categories of workers involved in this field. This would not only increase the level of general knowledge but also contribute to greater awareness in decision-making. In fact, FFs play a crucial role in advancing sustainability goals in the animal production sector by reducing feed and food competition and food waste, as highlighted by the European Commission Notice 133/02 of 2018 (64). However, a collaborative approach involving policymakers and key stakeholders in the field is essential to promote the use of FFs in farm animals and further reduce the environmental impact of the animal sector. Current trends in animal feed are focused on reducing the environmental impact of raw materials. This increasing sustainability in the animal production industry may therefore provide an encouraging scenario for sustainable animal feeds, including FFs.

## Data availability statement

The datasets presented in this article are not readily available because the data belongs to the University of Turin. Requests to access the datasets should be directed to corresponding author.

## Ethics statement

The studies involving humans were approved by the Ethical Committee of the Department of Veterinary Sciences from the University of Turin, approval n.0169873. The studies were conducted in accordance with the local legislation and institutional requirements. The participants provided their written informed consent to participate in this study. Written informed consent was obtained from the individual(s) for the publication of any potentially identifiable images or data included in this article.

## Author contributions

ED: Investigation, Methodology, Writing – Original Draft. KS: Writing – Original Draft. RO: Conceptualization, Writing – Review & Editing. SM: Writing – Review & Editing. VM: Writing – Review & Editing. AG: Conceptualization, Writing – Review & Editing. LO: Writing – Review & Editing. AS: Supervision, Writing – Review & Editing. FM: Writing – Review & Editing. JM-G: Writing – Review & Editing, Formal analysis, Visualization, Data curation. CF: Funding acquisition, Resources, Project administration, Writing – Review & Editing.
