# Supplementary material for: Influence of age, gender, and willingness to adopt former foodstuffs on the perception of Italian farm animal veterinarians
Source: Front Vet Sci. 2024 Jun 6;11:1396807. doi: 10.3389/fvets.2024.1396807 (PMC11188774; doi:10.3389/fvets.2024.1396807)
Supplement: Supplementary file 1 [file Table_1.PDF]

Supplementary Tables

Supplementary Table 1. Correlation plot showing Spearman's correlation between perceptions towards former foodstuffs as feed. Figures are labelled according to willingness of participants to try FFs as feed **A)** Willing to adopt and **B)** Unwilling to adopt

Supplementary Table 1.

A)

	Economic advantage		Feed consistency		Environmental sustainability		Positive social implications		Product availability		Antioxidant properties		Vitamin content		Supply of by-pass protein	
	p-value	rho	*p*-value	rho	*p*-value	rho	*p*-value	rho	*p*-value	rho	*p*-value	rho	*p*-value	rho	*p*-value	rho
**Consistency of the composition**	0.0002	0.3070														
**Environmental sustainability**	0.0000	0.4723	0.0013	0.2687												
**Social compliance**	0.0000	0.4671	0.0003	0.2992	0.0000	0.7788										
**Product availability**	0.0000	0.4186	0.0000	0.4857	0.0009	0.2774	0.0000	0.4007								
**Antioxidant properties**	0.0754	-0.1508	0.5232	0.0544	0.5980	0.0449	0.7617	0.0259	0.4771	0.0606						
**Vitamin content**	0.0728	-0.1521	0.8414	0.0171	0.7589	-0.0262	0.6383	-0.0401	0.4195	0.0688	0.0000	0.7690				
**Supply of by-pass protein**	0.7435	0.0279	0.9527	-0.0051	0.3384	0.0815	0.4687	0.0617	0.0386	0.1751	0.0000	0.5006	0.0000	0.5769		
**Digestibility and energy intake**	0.0396	0.1742	0.1515	0.1219	0.0051	0.2355	0.0904	0.1436	0.0090	0.2201	0.0001	0.3203	0.0000	0.3372	0.0000	0.3507

B)

	**Economic advantage**		**Feed consistency**		**Environmental sustainability**		**Positive social implications**		**Product availability**		**Antioxidant properties**		**Vitamin content**		**Supply of by-pass protein**	
	p-value	*r*-value	*p*-value	*r*-value	*p*-value	*r*-value	*p*-value	*r*-value	*p*-value	*r*-value	*p*-value	*r*-value	*p*-value	*r*-value	*p*-value	*r*-value
**Consistency of the composition**	0.4343	0.2031														
**Environmental sustainability**	0.0983	0.4143	0.3899	0.2229												
**Social compliance**	0.4986	0.1762	0.8151	-0.0613	0.0079	0.6201										
**Product availability**	0.1379	0.3751	0.4669	0.1892	0.4370	0.2019	0.0295	0.5277								
**Antioxidant properties**	0.2441	0.2988	0.5150	-0.1697	0.7554	0.0816	0.2266	0.3096	0.2276	0.3089						
**Vitamin content**	0.8564	0.0475	0.0693	-0.4509	0.1576	0.3585	0.0012	0.7187	0.1832	0.3390	0.0610	0.4634				
**Supply of by-pass protein**	0.2049	0.3238	0.3636	-0.2352	0.2008	0.3266	0.0116	0.5959	0.2487	0.2960	0.0000	0.8249	0.0014	0.7098		
**Digestibility and energy intake**	0.0194	0.5600	0.6324	-0.1251	0.1497	0.3650	0.0050	0.6473	0.1231	0.3886	0.0367	0.5094	0.0177	0.5669	0.0001	0.7995
