# Supplementary material for: Influence of age, gender, and willingness to adopt former foodstuffs on the perception of Italian farm animal veterinarians
Source: Front Vet Sci. 2024 Jun 6;11:1396807. doi: 10.3389/fvets.2024.1396807 (PMC11188774; doi:10.3389/fvets.2024.1396807)
Supplement: Supplementary file 2 [file Data_Sheet_1.pdf]

Appendix

English and Italian version of the survey

English version of the survey

Introductory informative section

Dear interviewee,
the questionnaire hereby proposed is a survey on the use of feed based on ex-food products in livestock feeding.
Former food products can be described as foods originally produced for human consumption (in accordance with EU Reg. 178/2002) and no longer intended for their original purpose for practical or logistical reasons, such as packaging defects; the product, however, does not present significant risks for the health of the animal or that of the consumer.
The survey is aimed at farm animal veterinarians who have been professionally practicing in Italy for at least 2 years, and who participate directly to the diets' planning (feed composition, origin, and quality of raw materials), and/or are involved in the feed production process (evaluation of the raw materials' quality, monitoring of the main production phases and check of the final product's quality).
By compiling it, the intention is to verify on multiple levels the perception and knowledge of a product from the feed industry which is gaining ever greater interest.
We kindly ask you to fill in all parts of the following questionnaire, otherwise it will not be possible to use it for statistical processing.
The data collected is totally anonymous and will not be used for profit.

Socio-demographic section

1. Select your gender:
   - Women
   - Men
   - Prefer not to say

2. Choose the age category you belong to:
   - 18-24
   - 25-30
   - 31-40
   - 41-50
   - 51-60
   - >61

3. Indicate the region you live in: *
   - North
   - Centre
   - South and Islands

4. Indicate your years of experience in the sector: * …..
5. Indicate the animal species you work with: *
   Please choose ONE of the options below
   a) Ruminants - if so, please specify by selecting all the options that fit:
   - Cattle
   - Ovine and caprine
   - Buffaloes

- Other: …

a) Swine
b) Poultry
c) Other: …

Former Foodstuffs section

6. Do you recommend the use of former foodstuffs as feed? *
   Please choose ONE of the options below
   a) Yes
   b) No
   c) Not sure

7. Indicate what are former foodstuffs products: *
   Please choose ONE of the options below. No right or wrong responses exist, only your personal opinion counts.
   a) Co-product of the agri-food supply chain whose production is impossible to avoid, but which has gained greater economic value (e.g. wheat).
   b) Food product no longer intended for human consumption due to non-compliance of an aesthetic-commercial nature.
   c) Waste generated during the production process.
   d) By-product unintentionally generated during the production process, characterized by commercial value (e.g. distiller).

8. How would you rate the importance of the following characteristics of former foodstuffs: *
   Please answer the following statements by circling ONE number beside each statement in an appropriate column. Circle any number to describe your personal degree of agreement. Make your ratings promptly, based on your first impression. No right or wrong responses exist, only your personal opinion counts

	5= Very important	4= Slightly important	3= Neutral	2= Slightly unimportant	1= Not important at all
Economic advantage					
Feed consistency					
Environmental sustainability					
Positive social implications					
Product availability					
Antioxidant properties					
Vitamin content					
Supply of by- pass protein					
Digestibility and energy intake					

9. Do you think that the use of former foodstuffs can represent a: *

Please answer the following statements by circling ONE number beside each statement in an appropriate column. Make your ratings promptly, based on your first impression. No right or wrong responses exist, only your personal opinion counts

Toxicological risk (presence of residues such as plastic, aluminium, etc, resulting from packaging)	Yes	No
Microbiological risk (bacteria, molds, mycotoxins)	Yes	No
Inaccuracy between actual and declared values reported on the label	Yes	No

Willingness to try section.

10. Are you willing to adopt former foodstuffs as feed in the future? *
    Please choose ONE of the options below
    a) Yes
    b) No

Original version of the survey

Sezione informativa introduttiva al questionario:

Caro/a intervistato/a,
il questionario che ti viene proposto è un sondaggio sull'utilizzo di mangimi a base di ex-prodotti alimentari nell'alimentazione zootecnica.
Gli ex-prodotti alimentari possono essere descritti come alimenti originariamente prodotti per il consumo umano (in accordo con il Reg. UE 178/2002) e non più destinati al proprio scopo originario per motivazioni di natura pratica o logistica, come per esempio difetti di confezionamento; il prodotto, tuttavia, non presenta rischi rilevanti per la salute dell'animale, né per quella del consumatore.
Il sondaggio è rivolto ai veterinari di animali da allevamento che esercitano attivamente in Italia da almeno 2 anni e che partecipano direttamente alla pianificazione delle diete (composizione dei mangimi, origine e qualità delle materie prime), e/o sono coinvolti nella processo di produzione dei mangimi (valutazione della qualità delle materie prime, monitoraggio delle principali fasi produttive e controllo della qualità del prodotto finale). Compilandolo si intende verificare a più livelli la percezione e la conoscenza di un prodotto dell'industria mangimistica che sta riscuotendo sempre maggiore interesse. Ti chiediamo gentilmente di compilare tutte le parti del seguente questionario, altrimenti non sarà possibile utilizzarlo per l'elaborazione statistica.
I dati raccolti sono totalmente anonimi, e non saranno utilizzati per fini di lucro.

Sezione socio-demografica

1. Indica il genere con cui ti identifichi:
   - Donna
   - Uomo
   - Preferisco non indicarlo

2. Indica la tua fascia d'età:
   - 18-24
   - 25-30
   - 31-40
   - 41-50
   - 51-60
   - >61

3. Indica la regione in cui vivi: *
   - Nord
   - Centro

- Sud e Isole

4. Indica il tuo livello di istruzione: *
    - Scuola primaria
    - Scuola secondaria
    - Istruzione universitaria o superiore

5. Indica i tuoi anni di esperienza nel settore: * …..
6. Indica le specie animali di cui ti occupi: *
   Per favore, seleziona solo una delle opzioni sottostanti
    a) Ruminanti - se selezionato, per favore specifica quali, selezionando tutte le opzioni applicabili:
        - Bovini
        - Ovicaprini
        - Bufali
        - Altro: …
    b) Suini
    c) Avicoli
    d) Altro: ….

Sezione Ex-prodotti alimentari

7. Raccomandi l'utilizzo di ex-prodotti alimentari come mangime? *
   Per favore, seleziona solo una delle opzioni sottostanti
   a) Si
   b) No
   c) Non so

8. Indica la definizione di ex-prodotti alimentari: *
   Per favore, seleziona solo UNA delle opzioni sottostanti. Non ci sono risposte corrette o sbagliate, conta solo la propria opinione personale
   a) Co-prodotto della filiera primaria la cui produzione è impossibile da evitare, ma che ha guadagnato un valore economico maggiore (es. grano)
   b) Prodotto alimentare non più destinato al consumo umano a causa di non conformità di natura estetica-commerciale
   c) Rifiuto generato durante il processo produttivo
   d) Sotto-prodotto generato durante il processo produttivo non intenzionalmente e caratterizzato da valore commerciale (es. distiller)

9. Come valuti l'importanza delle seguenti caratteristiche degli ex-prodotti alimentari: *
   Per favore, rispondi a questa domanda selezionando solo UN numero a fianco di ciascuna caratteristica, nell'apposita colonna. Seleziona il numero che meglio descrive il tuo grado personale di accordo. Effettua le tue valutazioni rapidamente, basandoti sulla prima impressione. Non ci sono risposte corrette o sbagliate, conta solo la propria opinione personale

	5= Di fondamentale importante	4= Molto importante	3= Abbastanza importante	2= Scarsamente importante	1= Per niente importante
Vantaggio economico					
Costanza della composizione					

Sostenibilità ambientale					
Implicazioni sociali positive					
Reperibilità del prodotto					
Proprietà antiossidanti					
Contenuto vitaminico					
Apporto di proteina by- pass					
Digeribilità e intake energetico					

10. Ritieni che l'utilizzo di ex-prodotti alimentari comporti un: *

    Per favore, rispondi a questa domanda selezionando solo UN numero a fianco di ciascuna caratteristica, nell'apposita colonna. Effettua le tue valutazioni rapidamente, basandoti sulla prima impressione. Non ci sono risposte corrette o sbagliate, conta solo la propria opinione personale

Rischio tossicologico (presenza di residui come plastica, alluminio, ecc, provenienti dall'imballaggio)	Si	No
Rischio microbiologico (batteri, muffe, micotossine)	Si	No
Discrepanza tra i valori nutrizionali effettivi e quelli riportati sul cartellino	Si	No

Sezione willingness to try

11. Saresti disposto a provare gli ex-prodotti alimentari come mangime in futuro? *

    Per favore, seleziona UNA delle opzioni sottostanti

    1. Si
    2. No
